# Can Standards and Regulations Keep Up With Health Technology?

**DOI:** 10.2196/mhealth.3918

**Published:** 2015-06-03

**Authors:** Christopher James Vincent, Gerrit Niezen, Aisling Ann O'Kane, Katarzyna Stawarz

**Affiliations:** ^1^UCL Interaction CentreUniversity College LondonLondonUnited Kingdom; ^2^Future Interaction Technologies LabDepartment of Computer ScienceSwansea UniversitySwansea, WalesUnited Kingdom

**Keywords:** governmental regulations, health services, medical devices, mHealth, mobile phones, open source initiative, software, standards, technology

## Abstract

Technology is changing at a rapid rate, opening up new possibilities within the health care domain. Advances such as open source hardware, personal medical devices, and mobile phone apps are creating opportunities for custom-made medical devices and personalized care. However, they also introduce new challenges in balancing the need for regulation (ensuring safety and performance) with the need to innovate flexibly and efficiently. Compared with the emergence of new technologies, health technology design standards and regulations evolve slowly, and therefore, it can be difficult to apply these standards to the latest developments. For example, current regulations may not be suitable for approaches involving open source hardware, an increasingly popular way to create medical devices in the maker community. Medical device standards may not be flexible enough when evaluating the usability of mobile medical devices that can be used in a multitude of different ways, outside of clinical settings. Similarly, while regulatory guidance has been updated to address the proliferation of health-related mobile phone apps, it can be hard to know if and when these regulations apply. In this viewpoint, we present three examples of novel medical technologies to illustrate the types of regulatory issues that arise in the current environment. We also suggest opportunities for support, such as advances in the way we review and monitor medical technologies.

## Introduction

In recent years, there has been a rapid, major, and continued advance in scientific discovery and technology proliferation that provides the means to support health care in new ways. For example, the percentage of UK adults who own a mobile phone has risen from 39% to 51% in just 1 year [1]. The proliferation of mobile phones and pervasiveness of health apps [2] allow patients to manage and track their health conditions on the go, which turns mobile phones into a tool for health-related behavior change [3]. This means that care can be provided outside of clinical settings [4,5] and technology can be used to address growing health care demands, such as an increasing prevalence of chronic conditions and aging populations [6].

A growing number of medical and health-related technologies becoming available can be adapted to support personal care, both in terms of customized hardware and software. For example, electronic devices are not only becoming ubiquitous, but are also easier to make; three-dimensional printers are becoming significantly cheaper (the market is predicted to grow by 500% in 5 years [7]). Three-dimensional printers are devices that create three-dimensional objects based on an electronic data source containing a three-dimensional model. As a result, these printers have opened up the possibility to produce custom-made medical devices as needed, where needed [8], which is sometimes referred to as “hyperlocal micro manufacturing” [9]. These types of advances will continue to provide solutions to health care problems that seemed near impossible to solve a decade ago, and they generate their own unique considerations about how these technologies fit into existing regulatory frameworks.

The need for regulation has long been established in the health care domain and has led to manufacturers considering safety during the design and evaluation of medical devices [10] (for a US perspective on ethical standards, see [11]). Medical device manufacturers often use medical device standards (eg, [12]) to guide their design and production processes and to comply with regulatory requirements. For example, the IEC 60601 [13] series puts in place requirements relating to safety and effectiveness, focusing on various aspects of the product (eg, electrical integrity, alarms). The IEC 62366 [14] standard describes a usability engineering process, to satisfy similar requirements, which is linked to a risk analysis standard (ISO 14971 [15]).

Unfortunately, novel and personal medical technologies do not always fit into the process specified in standards because they move away from what is currently and generally accepted as good practice to a situation in which there may be little or no precedent for comparison. Health care technology innovation may be hurt by the current regulatory system [16]. Sometimes standards do not provide sufficient design and evaluation criteria for novel technologies that differ significantly from equivalent predecessors; sometimes regulation may stifle innovation to the point where new technology cannot benefit the health care system (eg, through the time or cost constraints); however, sometimes existing systems may not be applied at all, or they are not applicable when it comes to modern technology.

In this paper, we open a discussion about the challenges to existing regulatory systems posed by novel and personal health care technologies. By presenting three examples that we have encountered as part of our research, we highlight some of the issues. First, we describe an open source infusion pump that raises questions about how to control the quality of custom-made medical devices. Next, we present our research on mobile medical devices that challenges the methods of evaluation set out in current medical usability standards. Finally, we discuss the design of a mobile phone app for medication adherence that may or may not be governed by the existing regulations. Although papers focusing on regulatory challenges have already been published (eg, [17]), we contribute to the discussion by introducing three case studies, outlining the issues with standards and regulations, and proposing ways to address these issues.

## Novel Health Care Technologies

### Overview

The following examples describe the tension between health care innovation and regulation. They come from research conducted as a part of the Computer-Human Interaction for Medical Devices (CHI + MED) project, focusing on developing tools to support safe and usable health technology (medical devices). The following section presents three technologies: open source hardware, mobile health care technologies, and health-related mobile phone apps. It describes the regulatory challenges that may be encountered in the development of these kinds of devices, and identifies opportunities for addressing these issues.

### Example 1: Open Source Medical Devices

#### Background

**Figure 1:**
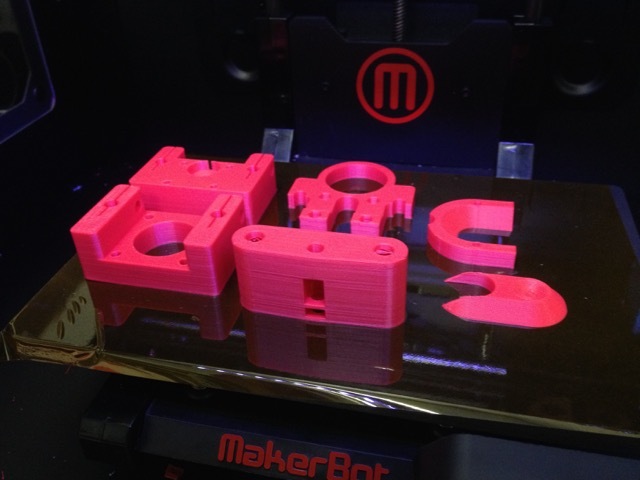
Three-dimensional printing technology (image credit Gerrit Niezen; image license CC-BY).

Open source hardware is an emerging business model where the design files of a product, including the circuit schematics, source code, and physical design, are made publicly available under a license so that anyone can study, modify, distribute, make, and sell the design or hardware based on that design. In recent years, three-dimensional printers have made possible a rapid production of customized medical devices [8], from fitted mechanical limbs [18] and mobile phone-connected microscopes [19] to parts for syringe pumps (as shown in Figure 1). Coupled with an open source approach, more can be achieved with less cost, because production can occur in-house, based on a freely available design.

Building on work of the Michigan Tech Open Sustainability Technology (MOST) group [20], we are demonstrating the process of building an open source syringe pump that implements design principles and interface guidelines published as part of the CHI + MED project. We are creating a complete open platform for further research and development [21]. Design files and software made public by MOST, under an open source license, are at the core of the project. The approach not only leads to economic savings through a reduction in the life-cycle cost [22], but also it allows others to improve on the design, share the improvements with others, and get rapid feedback from the end user. This openness can benefit multiple stakeholders and lead to effective technology and improved patient outcomes (for equivalent arguments relating to open source software, see [23]). It can also allow staff from hospital departments such as medical physics and clinical engineering to repair and customize their own devices, reducing a reliance on external providers.

#### Regulatory Challenges

The ability to modify someone else’s designs and the ease of rapid and unique production could interfere with formal quality-control processes, implicit in the existing medical device regulation. Although three-dimensional printing is a tool for prototyping and not for long-term use and reuse, it is possible to see how this technology could be used for the latter purpose.

Existing standards may not be practical as the documentation required for review and approval may be disproportionate when the design is limited to a very small number of production units. While the steps that are followed during the development and testing of medical devices are specified and controlled by standards (in the European Union, those listed in [12]), the process followed during the aforementioned activities may be ad hoc. For example, processes specified in medical device standards were created with traditional manufacturing process in mind. At a certain point, a design would be frozen and considered complete. This is not the case when devices can be continuously improved upon by the creator and others. The need for documentation and testing that closely adheres to standards may remove the flexibility that novel approaches bring. Repeated design changes and the requirement for oversight from a review body may be cumbersome on both sides. Although there are many advantages to realizing the benefits of existing process, systems need to be made agile and proportionate.

#### Opportunities

With an open source approach, there is an opportunity to share the rationale behind the design, the process used to derive the design, as well as the design itself. For example, online documentation tools such as wikis and version control software can be adapted to provide a better overview of the development workflow and process that has been followed. It is also possible to share evaluation results; if a component or design has evolved over time, knowing how and why this has happened could help those at distance understand the constraints of a solution. Through sharing and periodically updating these documents, duplication of effort can be avoided. For example, it does not always make sense for the same component to undergo the same testing by multiple parties. Moreover, documentation can be scrutinized by multiple specialists, without being confounded by the proprietary nature of “closed” solutions. Therefore, rather than requiring the same documentation as that for traditionally manufactured devices, regulation could involve transparent records of all components and changes made to those components, including the rationale and assumptions. This would help to support the quality of such devices without stifling innovation, with the onus being on those implementing a solution to check and review these documents.

### Example 2: Mobile Health Care Technologies and Home Use Devices

#### Background

Given the need to support care outside of clinical settings, technologies are also being developed to provide increasing autonomy and self-care. Our research on CHI + MED investigates how people use technology to manage their health care needs during their day-to-day lives. One such set of technologies includes devices used in the self-management of type 1 diabetes, a complex chronic condition that requires significant personal responsibility over a lifetime [24]. A common form of diabetes technology is the glucose meter that is used to measure blood glucose levels for everyday medication dose calculations, as well as for identifying high and low blood sugar levels, which are dangerous in the long term and potentially fatal in the short term, respectively [25]. Thanks to advances in technology and human factors engineering, glucose meters can be used by people with minimal or no training. They are easier to carry, easier to use, and can store results. This empowers people with diabetes and grants independence [26].

**Figure 2:**
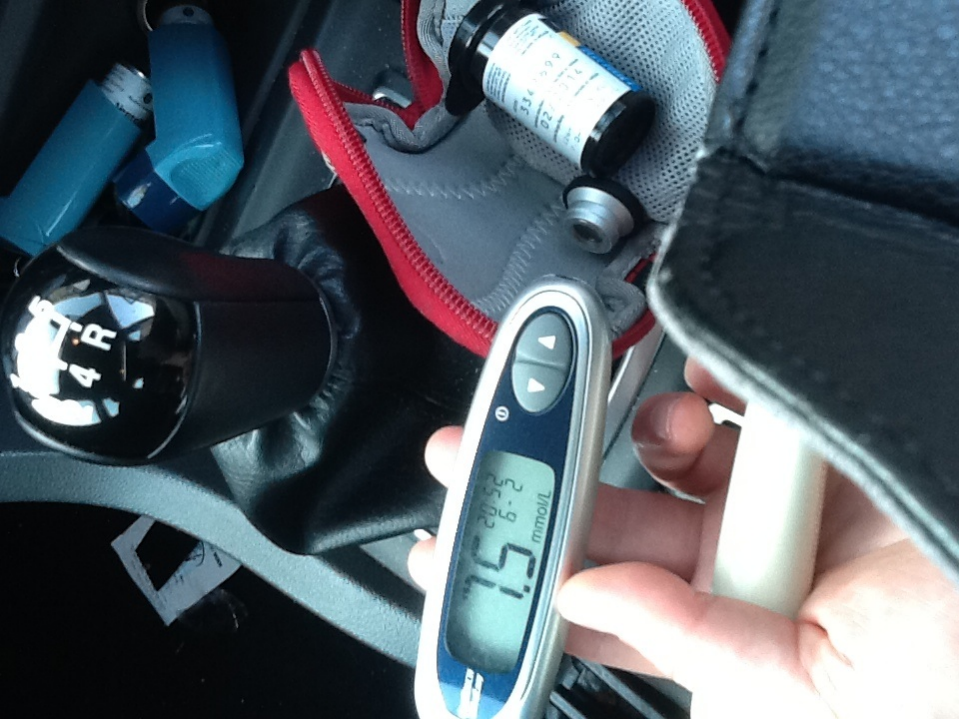
Everyday life and a glucose meter (image credit Aisling Ann O'Kane; image license CC-BY).

However, our work shows that complexities of everyday life such as people’s work life, romantic life, friendships, hobbies, travel, holidays [27], or whom they are with [28,29] impact on the use of these devices (Figure 2). Understanding these factors is incredibly complex [30], but necessary to ensure glucose meters are reliable and meet users needs. The problem is that the evaluation methods suggested by standards are not adequate in addressing everyday use, as they have been developed with a focus on technology used in clinical environments, where there is more certainty about the characteristics of the work place and levels of training.

#### Regulatory Challenges

Mobile health care technologies used for self-management, such as glucose meters used by people with diabetes, are medical devices and are regulated as such. Standard usability engineering process applies, such as IEC 62366 [14], for these cases.

These personal health care devices are used in the context of people’s everyday lives, yet the design and evaluation involves the same usability standards as medical devices found in hospitals. The definition of usability is the same (see [14]). The focus is on the device’s effectiveness, efficiency, ease of learning, and user satisfaction, in what is assumed to be a controlled context. This standardized usability evaluation practice makes it difficult to support the range of individual needs of users outside clinical environments: in their homes, on the go, as a part of their everyday life. This can be seen by accounts of the application of standard usability engineering process [31], such as IEC 62366 [14]. Although such standards are voluntary, and the techniques are illustrative, many companies feel that they have little option but to adhere to their content [32].

Although standards such as IEC 62366 [14] suggest taking context into account using techniques such as task analysis, contextual inquiry, functional analysis, testing in simulated clinical environments, and field testing, how they might be adapted or tailored to represent the unstable context of everyday life is not elaborated on. These methods may not capture the influences that a person’s life might have on the safe use and adoption of these devices, outside the confines of a hospital. Influences could include the emotional aspects of self-care or the social impact of bystanders, when using a device in front of others.

Likewise, it can be unmanageable to scope everyday health care technologies, for the purposes of making assumptions about their use. Pervasive technologies are used by all sorts of people, in all sorts of situations, and in all sorts of contexts. Individual differences are inevitable, and they have been shown to impact users’ experience and challenge the design and development of products [33]. This raises concerns for health care technologies where differences might result in safety risks.

These concerns can be addressed by limiting the scope to a certain user profile or context, but this may not be possible for medical technologies designed for a particular condition, not for a particular user group. Another approach is to configure products based on user needs, for example, allowing customization of the exterior shell [34] and/or allowing configuration of the user interface. This poses a dilemma for evaluation in that as the number of possible configurations increases, the burden associated with management, evaluation, and support also increases [35,36]. On the one hand, allowing for flexibility reduces the chance of nonadoption or noncompliance; on the other hand, complexity in the product adds to the resource required to develop and maintain it.

#### Opportunities

One option would be to increase emphasis on postmarket assessment, such as monitoring the use of equipment in context (including self-report), alongside conducting research to understand how users are really experiencing this type of equipment. Exploratory qualitative methods have been applied in other domains to focus on the situated use of interactive devices and they are also relevant here.

For instance, diary studies [37] involve users taking note of when, where, how, and why they use their device in their everyday life. They avoid the invasiveness of observation. Autoethnography, a form of self-study, is a quick and easy way to probe the everyday use of mobile medical devices [30]. Even though there has been progress toward using exploratory methods to investigate the context of use [38], standards are lacking in their treatment of situated user experience. Situated user experience relates to the notion that the localized context is an important factor in determining how people will experience and interact with technology. As it has been shown that context influences the use of pervasive health care technologies [30], testing technology away from this context (e.g. in a laboratory) does not anticipate how well the technology will meet the needs of the user. There is therefore an opportunity for standards to support consideration of a broader range of context, given the mobile nature of devices, and use outside of clinical settings. The inclusion of exploratory qualitative methods would allow for this by probing context of use and revealing of individual differences.

### Example 3: Health-Related Mobile Phone Apps

#### Background

Whereas personal medical technologies can provide benefits to those who require specific equipment, dedicated mobile phone apps have potential to be advantageous to almost anyone. People have access to thousands of free health-related mobile phone apps [39,40]. They range from behavior change apps supporting people who want to improve their health and well-being [3], for example, apps supporting smoking cessation (eg, SmokeFree28 [41]), or providing informed choice regarding alcohol intake (eg, Drinks Meter [42]), to apps focusing on specific conditions (eg, pain management [43]). They can be used to prevent forgetfulness (eg, medication reminder apps [44]) and general adherence support apps [45]. As people tend to keep their mobile phones close and hardly ever switch them off, health apps can provide useful functions at any time. They are always at hand to help track the on-going behavior.

Another (currently unreleased) example would be a software app to support oral contraception adherence. We are currently researching how mobile phone apps could be used to reduce unintentional nonadherence. This involves developing a medication reminder app that supports the formation of medication-taking habits and assists users as they search for the best way to embed medication taking into their daily routine; how this could be achieved is described in [44]. One of the major challenges to understanding the approach required to evaluate this type of software is the fact that it is not entirely clear whether an app should be regulated as a medical device.

#### Regulatory Challenges

During our research we have identified many issues concerning the certification of health-related apps. One example relates to the wording that is used to describe them. Many apps make medical claims and by doing so, could pose a serious public health issue, especially they are if ineffective or inaccurate [40]. Regulation is required and although guidance on health-related software and apps exists [46-49], it may not be clear whether such apps should be covered by regulations. Moreover, when considering the market as a whole, regulations may be bypassed and in some cases, ignored. In the European Union, in some cases, health-related apps fall under the control of the medical devices directive. In this scheme, assuming the software is not an accessory to a device, classification rules can treat medical standalone software as a comparatively low risk [49]:

“The classification rules were not written with software in mind. Due to the restrictive nature of Rules 9-11 of Annex IX to Directive 93/42/EEC, a large number of software devices therefore fall in Class I, where compliance is based on self-declaration, ie, no third party assessment.”

As a result, the product can be self-certified by the organization producing it. This raises concerns about the level of peer review and testing that this type of software receives.

In other cases, software can be used in a medical context, but not for a medical purpose, and not considered to be a medical device. For example, based on similar UK Medicines and Health Care Products Regulatory Agency (MHRA) advice, an app could use an accelerometer or gyroscope to detect falls in epileptic patients and therefore be regulated as a medical device. However, if the same technology is used to measure steps or detect whether an elderly person has got up from a chair or a bed in a social care context, the regulation would not apply [47]. This is important because it impacts on the type of testing that would apply (both in terms of usability and general safety and performance requirements), as well as the approach to monitoring the device in situ.

To help determine whether an app should be treated as a medical device, the MHRA has produced the following list of keywords and phrases that if used in the app’s description indicate that it should be regulated: *amplify, analysis, interpret, alarms, calculates, controls, converts, detects, diagnose, measures,* and *monitors* [47]. Take the example of an app that aims to send alerts to users to check whether they have taken their medication and records how many times they say they did not. Based on this information, it can suggest changes to the routine. Therefore, it could be said that the app *monitors* users and occasionally *alarms* when their behavior needs to be modified. Does it mean the app should be certified?

If the app is labeled as a tool for supporting *medication-taking habits*, then the answer is likely to be yes. However, if the wording changes to simply *habits*, then even though the functionality stays the same, does the answer become no? Such a small change in wording could be enough to avoid device regulations, but it might not even be needed. Some developers simply publish their health apps without worrying about regulations at all, whereas others add liability disclaimers to app descriptions [40]. Based on the US guidance relating to mobile medical applications [46], “Mobile apps that keep track of medications and provide user-configured reminders for improved medication adherence” are an example of “...mobile apps for which FDA intends to exercise enforcement discretion” [46].

Based on both US and UK frameworks, this type of app may or may not be regulated. This problem has been covered in recent UK media reports [50], where there are several examples of gray areas and products that sit on the boundary. In many respects there is not anything new about technologies that sit on the boundary between regulated and unregulated products. The concern is that the assumptions used to determine whether technology is regulated as a medical device may not reflect how the technology is actually used (eg, unregulated apps being used in a context when a regulated app is appropriate).

Of specific concern is the quality of the software code and process used during programming, which may be invisible once the software has been released. Even if there is intent to follow medical device standards, they may be difficult to realize in practice. For example, the medical device usability standard IEC 62366 [14] combines evaluation of safety and usability, which may be in opposition to each other [35]. Other standards may be insufficient when it comes to the testing of software: they may not require exhaustive testing, complete coverage, or proof that a solution is correct by construction [51]. This is evident in the number of software-related defects observed in medical device user interfaces [52].

#### Opportunities

Because of the intangible nature of apps and the fact that they can be easily updated in situ, assessment and classification at a single point in time may not be feasible or appropriate. Obiodu and Obiodu [40] suggest that one way to deal with the issue of certification might be producing evidence-based guidelines for designing health apps rather than trying to strictly regulate them. We agree with this point, but would see an opportunity to take this further. Rather than just relying on guidelines issued by authorities, patient groups could produce best practice guidelines for specific conditions and, by following the example of open hardware initiatives, publish them openly to encourage collaboration with other patient groups, app developers, and mobile phone manufacturers, who are already starting to release health care kits [53].

## Conclusions

For medical technology, standards and regulations are needed to ensure safety, protect the public, and guarantee that products are fit for purpose. However, in the context of novel and personal medical technologies, the current approach to regulation is not only infeasible and difficult to enforce, but also work against health care innovation. Given that it is inevitable that three-dimensional-printed components, mobile devices, and apps will be used to support and deliver health care, as well as have impact on medical practice, regulators may need to rethink their approach.

Based on our work, we have presented the benefits of new technologies and personal medical devices. In many cases, growing pressure on health services makes their introduction inevitable. At the same time, we have outlined some of the regulatory challenges. For example, by allowing for rapid manufacturing of bespoke components, three-dimensional printing raises concerns regarding quality control; the standards underpinning the usability of personal mobile medical devices are not enough to guarantee the “design meets users’ needs” concept; in addition, mobile phone apps may or may not be certified, depending on how a product is described. These challenges open up new possibilities and encourage new ways of thinking.

The health care domain is not the only one feeling the impact of these technologies. The situation resembles the issues with touch-screen tablets being used in the office environment. Although office work and office equipment are regulated (eg, computer workstations), the health and safety regulations are unlikely to apply to tablet computers when it is not possible to easily control how or where they are being used [54]. Rather than trying to regulate them, different, more flexible approaches are needed. For example, by shifting focus away from the introduction of technology, and toward educating users about the implications of using it (eg, raising awareness of human factors), we allow those in different environments to make sure their technology is safe and fits their needs. Although due diligence occurs during the design of technology, continual research and review aims to tailor the properties of equipment with the needs of users and characteristics of the work environment.

The same could apply to novel and personal health care technologies. When it comes to testing for usability, we cannot predict every possible combination of user and usage before the deployment of technology. The alternative is to conduct research into how equipment is really used. We then realize that improvement will occur when a device is in situ, and this will be specific to a given context. As we can rapidly iterate a design, we can continually improve and share the benefit of this improvement. Much of the existing guidance concerning safety and usability needs updating to accommodate this approach. There also needs to be a consideration of how adequate levels of safety can be guaranteed, without making it prohibitive for small organizations to create products with relatively short life cycles. The problem with the existing approach to regulation is that historically, those producing technology would be likely to stay in business for extended periods, compared with the hobbyists and small organizations producing apps, who would rapidly develop and release technology, but then may not be in place to support it in the future. In the past, we had traceability and accountability, whereas in the present we have little recompense if something goes wrong.

We suggest ways of addressing these challenges, such as publishing documentation and making it openly available to review, therefore increasing transparency; adding situated methods to usability standards to cover people's everyday use of personal health technology, and allowing patient groups to review mobile phone apps, draft their own guidelines, and collaborate with each other and with app developers. This would help to ensure that patients’ needs are met. Realizing a code of practice for app developers, such as PAS 277 [55], would help to build confidence. There is also an opportunity to educate those buying and using such technology on requirements relating to safety and usability.

If there is a need to comply with medical device software process standards (eg, IEC 62304 [56]), there have been recent developments in guidance. There are now worked examples of assessment process (ISO 33030 [57]); and support for process tailored to the safety class of the software (IEC/TR 80002-3 [58]). There are groups such as Medi SPICE [59].

This viewpoint represents a series of observations from our own research on the challenges of regulating health technology. We hope to start a discussion about the obstacles and opportunities in addressing the design of novel technologies within regulatory frameworks. Given the need to address the increasing pressures on health services, this discussion is urgently required. Future research could apply a structured methodology to review this context and a case study approach [60], to articulate practical, balanced, and proportionate approaches in line with this discussion.

## Abbreviations

CHI+MED: Computer-Human Interaction for Medical Devices
MHRA: Medicines and Health Care Products Regulatory Agency
MOST: Michigan Tech Open Sustainability
